# The Influence of New Colored Light Stimulation Methods on Heart Rate Variability, Temperature, and Well-Being: Results of a Pilot Study in Humans

**DOI:** 10.1155/2013/674183

**Published:** 2013-11-28

**Authors:** Daniela Litscher, Lu Wang, Ingrid Gaischek, Gerhard Litscher

**Affiliations:** Stronach Research Unit for Complementary and Integrative Laser Medicine, Research Unit of Biomedical Engineering in Anesthesia and Intensive Care Medicine, and TCM Research Center Graz, Medical University of Graz, Auenbruggerplatz 29, 8036 Graz, Austria

## Abstract

Changes of light intensity of different colors can shift many physiological parameters and conditions like melatonin, alertness, body temperature, heart rate (HR), and heart rate variability (HRV). The aim of this pilot study was to investigate acute temperature, HR, HRV, and state of mind reactivities after illumination with red (631 nm) and blue (456 nm) light (illuminance 140 lux for both). Seven healthy volunteers (5 females, 2 males; mean age ± SD 34.1 ± 11.9 years) were investigated at the Medical University of Graz, using new color light panels. Significant decreases were found only after 10 min blue light stimulation in nose temperature (*P* = 0.046), HR (*P* < 0.05), and total HRV (*P* = 0.029), in association with a significant alteration of the emotional state (stress level score, *P* = 0.006). However, red light stimulation of the same persons did not induce the same effects in these parameters. The effect of blue light as environmental stimulation on human health is not clarified in detail and needs further investigations.

## 1. Introduction 

Human beings are very sensitive to light exposure, and changes of light intensity can shift many physiological parameters like melatonin, alertness, body temperature, heart rate (HR), and heart rate variability (HRV) [1]. In this context the effects of colored light have been investigated in few scientific studies [2–4], in addition to the alterations based on changes of white and bright light [5–7].

In previous investigations it has been found that colored light can influence the HRV within minutes and that the effects of individual colors can be differentiated by HRV [3]. It has also been reported that the HRV ratio of low frequency to high frequency (LF/HF) was decreased after illumination with so-called “cold colors” [2].

The goal of the present pilot study was to investigate acute HR, HRV, temperature, and state of mind reactivities after illumination with differently colored light (red and blue) during daytime in healthy volunteers with closed eyes.

## 2. Materials and Methods

### 2.1. Subjects

Seven healthy volunteers (5 females, 2 males; mean age ± SD 34.1 ± 11.9 years; range 23–55 years) were investigated at the Medical University of Graz. None of the subjects was under the influence of centrally active medication, and one had a history of heart or cerebrovascular disease, respiratory or neurological problems, or hypertension. All volunteers gave oral informed consent, and the study was carried out in compliance with the Declaration of Helsinki.

### 2.2. New Colored Light Stimulation Methods

**Figure 1:**
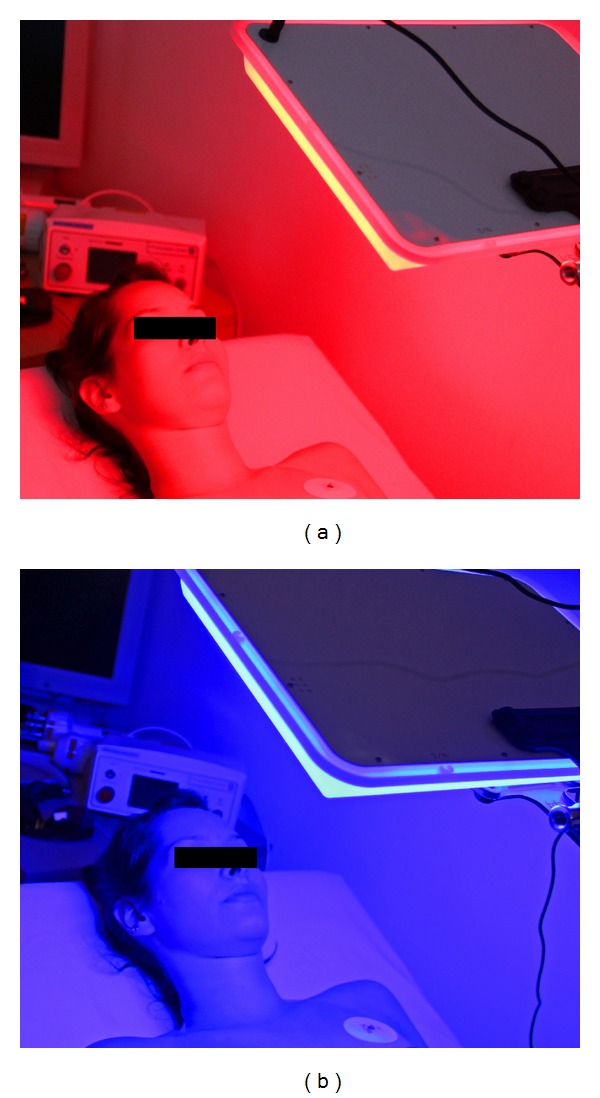
Stimulation with red (approx. 631 nm; (a)) and blue (approx. 456 nm; (b)) color panels (approx. 140 lux).

Two color light panels (collaxx, mse elektronik, Frankenburg, Austria) were used in this study (see Figures 1(a) and 1(b)).

**Figure 2:**
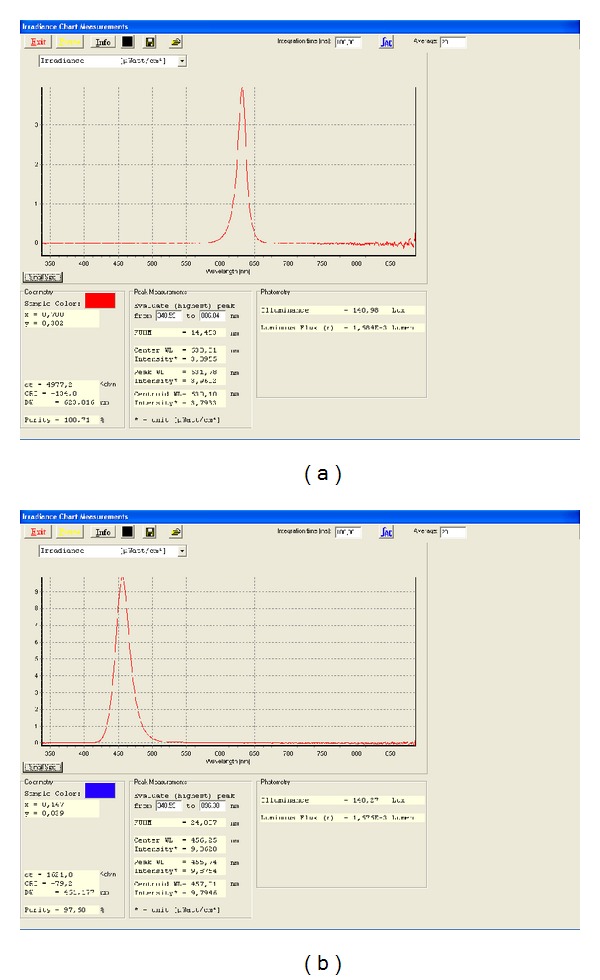
Colorimetry and photometry of the two light panels used in the study. (a) 631 nm, red; (b) 456 nm, blue.

Both colors had almost the same illuminance (red: 140.98 lux, and blue: 140.27 lux, measured at a distance of 40 cm).  Figure 2 shows the spectra of the two colors. In addition, the dominant wavelengths (DWred = 623.0 nm and DWblue = 461.2 nm) are indicated. DW is the wavelength which is important for the sensitivity of the human eye. The peak wavelength differs from the DW because both colors, red and blue, are located at the (opposite) margins of the visible light spectrum, and the human eye's sensitivity for brightness is drastically reduced in these regions.

### 2.3. Temperature Measurements

The temperature measurements were performed using a Flir i7 (Flir Systems, Wilsonville, USA) infrared camera which operates at a wavelength range from 7.5–13 *μ*m. The focal distance of the infrared lens is *f* = 6.8 mm. The temperature measurement range is between −20°C and +250°C. Its accuracy lies at ±2% of the reading. Sensitivity is <0.1°C at 30°C, and the infrared resolution is 140 × 140 pixels. The system is ready for use in 15–20 seconds. We chose the forehead and the tip of the nose as locations for the thermographic measurements. Both areas were measured during illumination and also during the control phases before and after illumination.

### 2.4. Electrocardiographic Measurements

Electrocardiogram (ECG) is registered using three adhesive electrodes (Skintact Premier F-55; Leonhard Lang GmbH, Innsbruck, Austria) which are applied to the chest. The duration of RR-intervals is measured during time periods of 5 min, and on spectral analysis basis HRV is determined.

A medilog AR12 HRV (Huntleigh Healthcare, Cardiff, United Kingdom) system is used. The system has a sampling rate of 4096 Hz [8], and the raw data are stored on a memory card. Mean HR, total HRV, and the LF/HF ratio of HRV were chosen as preliminary electrocardiographic evaluation parameters, as such being recommended by the Task Force of the European Society of Cardiology and the North American Society of Pacing and Electrophysiology [9].

### 2.5. Procedure

**Figure 3:**
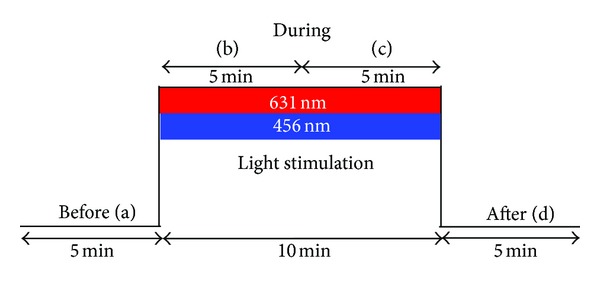
Experimental protocol.

The experiment used a repeated-measures design with two different light conditions (see Figure 3) and took place during daytime (between 09:00 and 11:00) in July 2013 (room temperature: 28–30°C). Every volunteer completed the investigation. The persons were lying on a bed with closed eyes in a special lab of the Medical University of Graz, and the measurements started after a resting period of 5 minutes. The volunteers were exposed to the two differently colored stimulations for 10 minutes in randomized order. Between the two different stimulation modalities there was a resting period of 10 min. This duration was chosen for practical reasons (e.g., HR should return to baseline values in this time). HRV was measured during stimulation ((b) and (c) in Figure 3), and also for 5 min before (a) and after (d) illumination with red or blue light. After exposure to the red and blue light, respectively, the volunteers reported their state of mind for each modality. These reports were categorized on a scale from 0 to 10 (0: positive, comfortable, and relaxed; 10: negative, uncomfortable, and anxious).

### 2.6. Statistical Analysis

Data were analyzed using SigmaPlot 12.0 software (Systat Software Inc., Chicago, USA). Testing was performed with one way repeated measures ANOVA and Holm-Sidak test. In addition, paired *t*-test was used. The data are graphically presented as mean ± SE (standard error). The criterion for significance was *P* < 0.05.

## 3. Results

**Figure 4:**
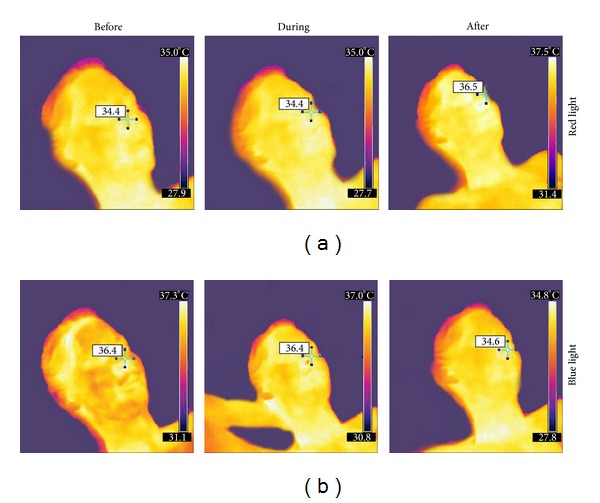
Six thermal images from a 23-year-old healthy female volunteer before, during and after red (a) and blue (b) light stimulation with closed eyes. Note the increase of the temperature of the nose after red and also the decrease after blue light irradiation.

A typical example of the results of thermal imaging is shown in Figure 4. The example demonstrates the face of a 23-year-old female. The room temperature was 30°C. It is interesting that after red light stimulation the temperature of the nose (marker in Figure 4) increases from 34.4°C to 36.5°C. In contrast, after blue light stimulation the temperature of the nose of the same person decreases from 36.4°C to 34.6°C.

**Figure 5:**
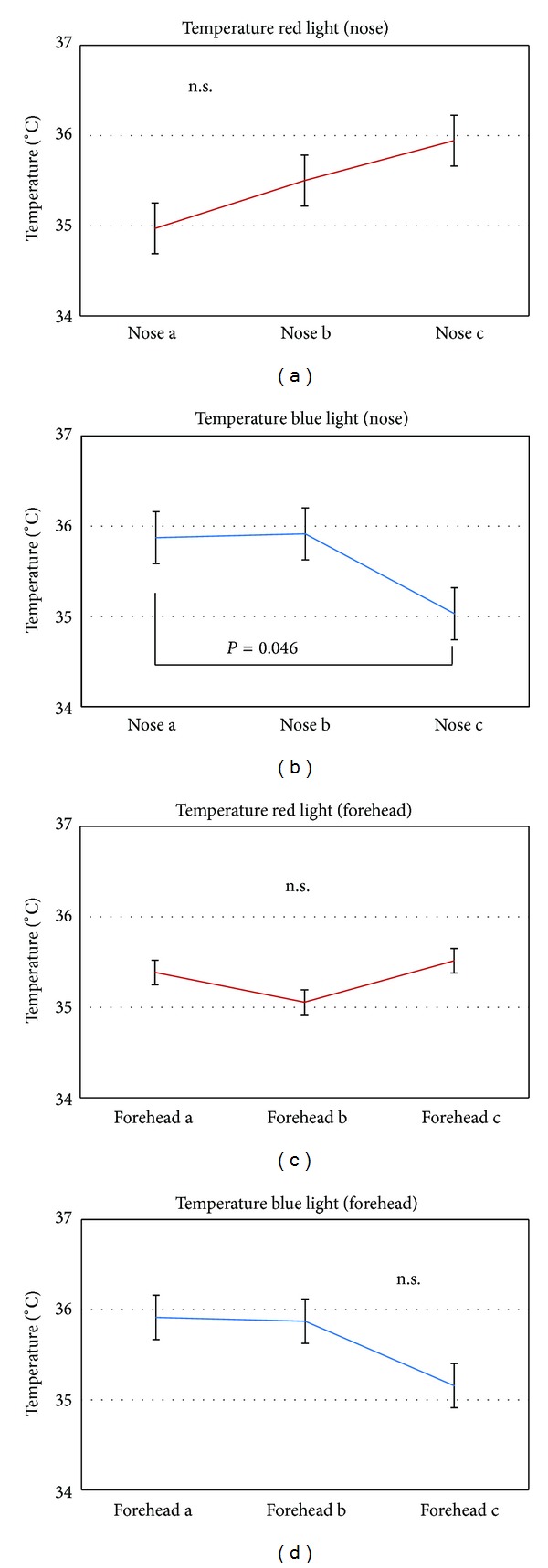
Temperature values at the nose and forehead before (a), during (b), and after (c) irradiation with red and blue light. Note the significant decrease of the temperature at the nose after blue light stimulation.

Figure 5 summarizes data extracted from the thermal images. Blue light decreases temperature in most of the volunteers (measured at the nose and forehead), whereas red light leads to a slight increase.

**Figure 6:**
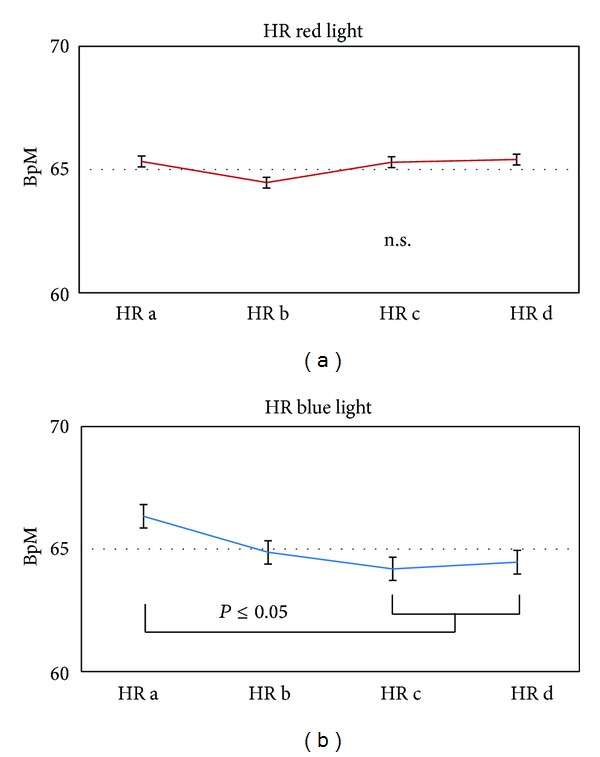
Graphics displaying the changes in mean heart rate (HR) of the 7 subjects receiving red light stimulation (a) and the same persons receiving blue light stimulation (b). HR decreased significantly only following stimulation with a wavelength of 456 nm.

**Figure 7:**
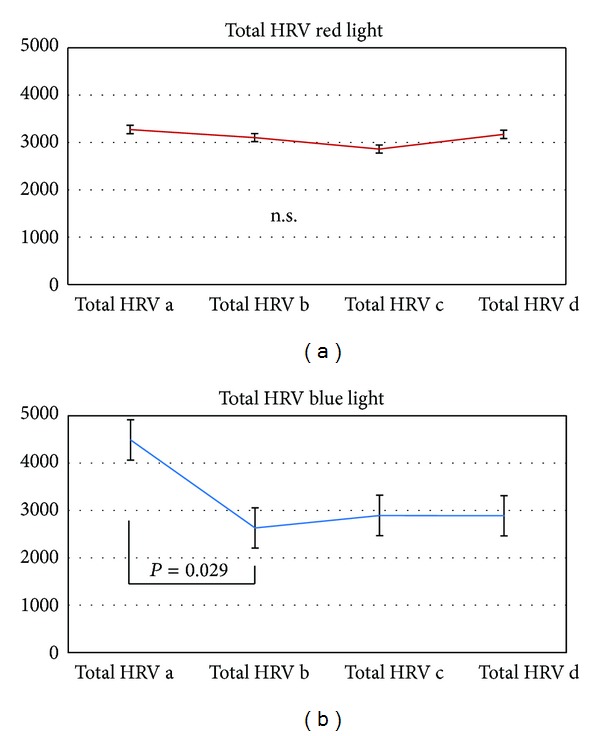
Changes in total heart rate variability (HRV). Blue light (right) stimulation induced significant stimulation-related changes in total HRV in the seven subjects investigated in this study. No significant changes were found for red light stimulation.

Figures 6 and 7 show the mean HR and total HRV from the ECG recordings of altogether seven healthy volunteers during the four measurement phases (a–d). There was a slight but not significant decrease in HR after stimulation onset with red light. However, in the blue light session HR decreased significantly (*P* < 0.05) during the second half of the stimulation phase and also in the 5 min period afterwards (Figure 6).

In addition to HR, HRV also showed significant (*P* = 0.029) alterations in the blue light session.

**Figure 8:**
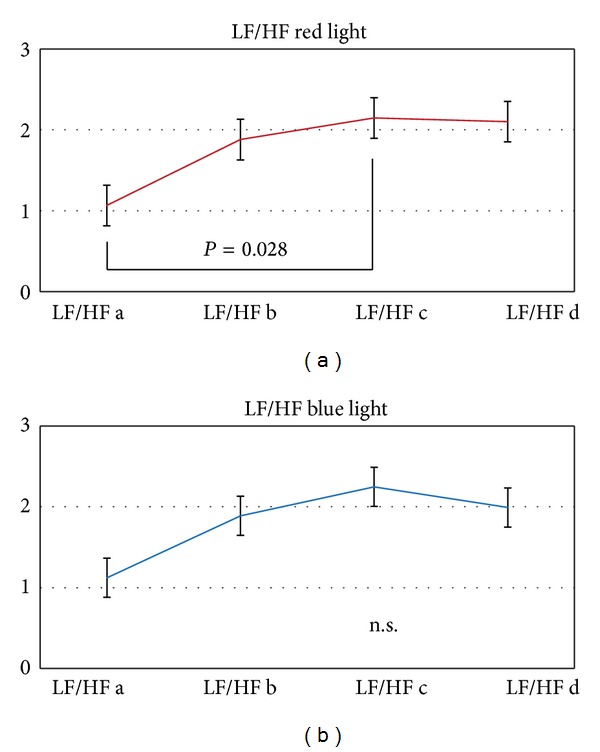
The low frequency (LF)/high frequency (HF) ratio changed significantly during red light stimulation (*P* = 0.028).

Continuous HRV monitoring also showed significant alterations in the LF/HF ratio during red light stimulation (see Figure 8).

**Figure 9:**
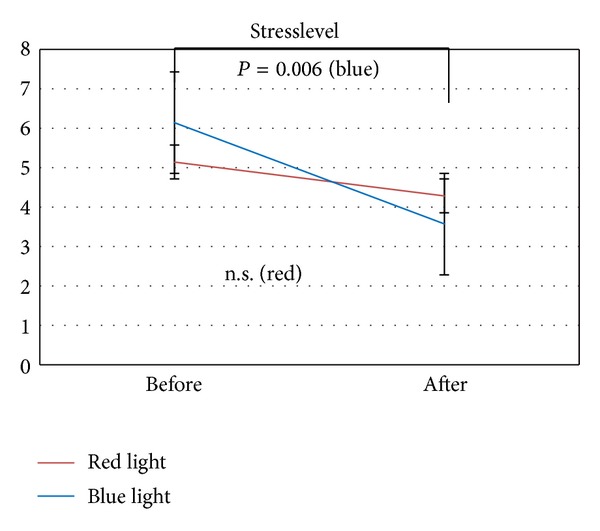
Stress level score evaluated in the 7 subjects.

The results of the state of mind questionnaires are summarized in Figure 9. A significant (*P* = 0.006) decrease in the sense of a positive effect of well-being was found after blue light stimulation.

## 4. Discussion

Light plays a central role in life. Without sunlight there is no life on earth. Effects of light stimulation and light therapy on autonomic functions (e.g., body temperature, HR, or HRV) were already investigated in several human studies [5, 10, 11]. To the best of our knowledge, simultaneous recording of the three parameters has never been performed extensively during red and blue light stimulation. However, the results of this preliminary study should be regarded as those of a pilot study and thus require cautious interpretation.

The temperature results of the present study demonstrate that illumination with blue light for 10 minutes evoked significant changes in regional temperature at the nose. In addition, significant HR and total HRV reactivities were associated with alterations of the emotional state of the participants (stress level score). However, the red light stimulation did not induce significant changes in temperature, HR, and total HRV in the same persons. Furthermore the stress level score did not show significant alterations after red light stimulation with the same illuminance and distance to the eye.

Specific effects of different light colors have been reported in several studies. Exposure to low intensity blue light can have an acute alerting effect without melatonin suppression [12]. Other authors reported that red light activates avoidance, whereas blue light enhances approach [13], but it is also stated that the associated psychological processes have not been fully explored [2]. Our study is one of the first to demonstrate interactions between different parameters (temperature, HR, HRV, and a score) during and after exposure to two different light colors in the same persons during a relatively short time and during nearly identical steady-state laboratory conditions. This study design therefore minimizes intra- and interindividual subject variability.

There are reports about changes of nose temperature in evidence-based complementary studies, for example, after acupuncture stimulation. Zhang et al. [14] already showed in 1991 that nose temperature can be lowered immediately after acupuncture. This is also described in theories of ancient Chinese books [14].

Indicators of the functional state of the autonomic nervous system like temperature, HR, or HRV were also investigated by Suter and Kistler in the last century [15]. The authors pointed out the importance of studying basic regulatory mechanisms which are fundamental for most treatments in complementary medicine.

The sensitivity of HRV as an index of effective emotion regulation was demonstrated furthermore by Elliot et al. [16]. Participants who were exposed to red light (versus a control color) exhibited a decrease in HF-HRV, and this result was associated with worse cognitive performance [16].

In addition to HR and total HRV we also calculated the LF/HF ratio. Another study reported that this ratio was decreased after illumination with “cold” colors [17]. We could not confirm these results; in contrast, there was a slight but insignificant increase of the LF/HF ratio also under blue light stimulation. However, previous reports also mentioned that it is important to take the individual emotional state into account for such investigations [2].

Our results showed that the blue light altered total HRV, whereas the red light altered the LF/HF ratio. Maybe different pathways and activations in the brain are responsible for these results, however, our study design does not allow conclusions concerning the underlying mechanisms.

In addition it is also interesting that the changes in HRV during blue light stimulation appeared before HR changed significantly. This demonstrates that the HR changes per se were not the main factor influencing HRV.

The influence of sound and light on HRV was also demonstrated in several previous studies [18]. Authors from Japan found that the cardiac parasympathetic nervous activity during auditory excitation increases with elimination of visual stimuli and tends to be more pronounced in females than in males. In our study no comparison between females and males was performed due to the small sample size.

At this point it has to be mentioned that there are some limitations of this study. Firstly, as already stated, the number of persons included in the study is very small. Nevertheless there were significant changes in different parameters which made a common interpretation meaningful. Secondly, a possible response bias is always inherent in self-report information like the stress level score in our study, and thirdly, due to the small sample size, the baselines values of the temperature differed at the beginning of the blue and red light stimulation, respectively.

In conclusion of our study, blue light stimulation induced more significant effects in quantitative measurement parameters of the autonomic nervous system in comparison to red light stimulation with nearly the same illuminance and distance from the eye. The results also show that the objective, measurable effects were associated with subjective impressions of the test persons. However, it should also be stated very clearly that the different effects of colored light as environmental stimulation of human health are not clarified in detail at the moment and thus this topic deserves further studies.
